# The impact of perceived risk of online takeout packaging and the moderating role of educational level

**DOI:** 10.1057/s41599-023-01732-9

**Published:** 2023-05-09

**Authors:** Meiwen Guo, Liang Wu, Cheng Ling Tan, Jun-Hwa Cheah, Yuhanis Abdul Aziz, Jianping Peng, Chun-Hung Chiu, Rongwei Ren

**Affiliations:** 1 School of Management, Guangzhou Xinhua University, Guangzhou, China; 2 Graduate School of Business, Universiti Sains Malaysia, George Town, Penang Malaysia; 3 Entrepreneurship Center, Sun Yat-sen University, Guangzhou, China; 4 School of Business and Economics, Universiti Putra Malaysia, Serdang, Selangor Malaysia; 5 Department of Information Technology & Management, Daffodil International University, Dhaka, Bangladesh; 6 Norwich Business School, University of East Anglia, Norwich, UK; 7 School of Marxism, Sun Yat-sen University, Guangzhou, China; 8 School of Business, Sun Yat-sen University, Guangzhou, China

**Keywords:** Business and management, Health humanities

## Abstract

With the rapid development of e-commerce and the impact of COVID-19, online takeout has become the first choice of more and more consumers. Previous research has indicated that food packaging is of great significance to marketing performance, yet very little is known about the mechanisms through which food packaging pollution risk affects online takeout consumption. This study proposes an expanded model of the Theory of Planned Behavior (TPB) by incorporating the Concept of Perceived Risk (CPR) to analyze the mechanism of consumers’ packaging pollution risk perception (PPRP) on their purchasing intention toward online takeout. Online survey was performed to collect data from 336 valid respondents in China, which was analyzed using structural equation modeling. The research findings verify the effectiveness of the TPB in the context of Chinese online takeout. Notably, the PPRP of online takeout was found to have a significant negative impact on consumers’ attitudes, subjective norms, and perceived behavioral control (PBC). It was also confirmed that consumers’ attitudes, subjective norms, and PBC regarding online takeout partially mediate the negative relationship between PPRP and purchase intention. In addition, the findings corroborate the granular nuances among three groups concerning consumers’ education level. The results do not only provide suggestions to the online takeout industry but also contribute theoretical value and practical significance for the improvement of sustainable food consumption.

## Introduction

Undoubtedly, the online takeout industry is developing rapidly, especially in view of the current epidemic that has bolstered people’s preference to order food online to avoid physical contact (Sohu.com, 2020; Kumar and Shah, 2021; Kumar et al., 2021; Guo et al., 2021a, 2021b), and grocery retailer actively explore the online channel to expand their market share (Dominici et al., 2021). In fact, Chinese takeout platforms Meituan and Eleme saw an increase of 7.5% in revenues from 2019 to 2020. At US $13.71 billion and US $3.95 billion in revenues, respectively, they ranked first and second in the top 10 takeout platforms in the world in 2020 (Alibaba, 2020). However, the accelerated growth of the online takeout industry has also exposed several problems. As more people turn to online shopping and takeout services, a large number of goods are packaged in plastic and sent to residences; the consequent plastic pollution carries huge potential risks for the environment and human health (Organization for Economic Co-operation and Development, 2018; Molloy et al., 2022). According to the United Nations Environment Program, the negative spillover effects of plastic waste on fisheries, tourism, and marine transportation are estimated to total US $40 billion per year (Souhu.com, 2020). In response to this, countries all over the world have actively introduced laws to prevent plastic pollution from causing greater harm to mankind (cn-hw.net, 2020). For example, as of July 1, 2019, restaurants in New York no longer use disposable foam lunch boxes, while in June 2019, 34 of the 55 countries on the African continent issued relevant decrees to prohibit or tax the use of disposable plastic bags (cn-hw.net, 2020). Nevertheless, the epidemic has intensified people’s tendency to rely on the use of takeout far more than before, which produces unprecedented amounts of plastic waste and places more pressure on prior regulations against plastic pollution.

At the same time, plastic pollution caused by the takeout industry has not been fully researched, though abundant academic advancements have been made in the study of municipal solid waste management, including the impact of urban garbage on the environment (Barnes, 2019; Razzaq et al., 2021; Zhang et al., 2022), garbage recovery and disposal (Giordano et al., 2021; Bai and Lin, 2022), and garbage supervision and valuation (Khan et al., 2022). With the development of online takeout platforms, the scale of online takeout is becoming larger than ever, resulting in more takeout packages and more harm to the environment and human health—these issues need to be paid much more empirical attention (Molloy et al., 2022).

With regard to consumers’ takeout purchase behavior, many scholars have focused on how to improve consumer service quality, such as through large-scale group decision-making (Xuan, 2022), chatbots (Leung and Wen, 2020), and the reduction of online product uncertainty (Youngsoo and Ramayya, 2015). In contrast, few have raised concerns about the takeout risks emerging from the epidemic, such as environmental and human health risks (Liao et al., 2021; Arunan and Crawford, 2021; Xie et al., 2021; Schuermann and Woo, 2022). Under the influence of the COVID-19 epidemic, consumers have begun to pay more attention to the impact of their consumption on the environment and human health. For instance, many consumers are aware of the environmental repercussions of food packages and are willing to use healthier eco-friendly packaging (Lazzarini et al., 2016), such as recyclable packaging, glass packaging, paper packaging, and smart packaging (Holman et al., 2018; Muller and Schmid, 2019). Furthermore, consumers are seen to accept biodegradable food packaging (Lange, 2017; Moshood et al., 2022) even though it is costlier (Fernqvist et al., 2015; Granato et al., 2022).

Therefore, the first research gap this study sought to address is whether the perceived risk of online takeout packages influences consumers’ purchase intention towards online takeout. Despite consumers’ growing awareness of the environmental repercussions of food packages, their understanding of the risks of food packaging remains inadequate. They are more objective about the environmental impacts of commonly used paper and metal packaging, yet they underestimate the risks associated with plastic packaging while overestimating the benefits of biodegradable plastic packaging. In essence, consumers still lack knowledge about the environmental impact of food packaging. Even so, the initial motivation of consumers is to avoid pollution as much as possible, regardless of the outcome of their behavior. This means that even if consumers’ purchase behavior is based on environmental protection, it still results in environmental risk in many cases (Otto et al., 2021). Consequently, it is necessary to verify the negative effect of the perceived risk of takeout packaging on consumers’ purchase intention, which is the first gap bridged by this study.

Furthermore, the second gap this study managed to close is how the perceived risk of online takeout packages impacts the consumers’ purchase intention towards online takeout. From the perspective of the Theory of Planned Behavior (TPB), online takeout purchase intention is positively affected by consumers’ attitudes, subjective norms, and perceived behavioral control (PBC). Many studies have applied the TPB to the area of sustainable consumption, such as organic food purchase intention (Nagaraj, 2021). In addition, attitude, subjective norms, and PBC have been found to mediate the effect of environmental concerns on green product purchase intention (Paul et al., 2016). Despite its adoption in various contexts, the TPB usually considers positive antecedents of intention (Yeh et al., 2021). Notably, there is some research in the field of renewable energy/energy efficiency technology consumer behavior (Tanveer et al., 2021; Poier, 2021; Busic-Sontic and Brick, 2018; Pires et al., 2004), which incorporated risk factors into TPB. Grounded on these research findings, this study identified online takeout packaging pollution as the risk driver negatively informing TPB. In other words, consumers face a trade-off between environmental pollution and the convenience brought by takeout. The concept of perceived risk (CPR) posits that consumers typically take measures to avoid risks. For example, upon realizing that online takeout brings risks to the environment, consumers are likely to reduce their consumption intention. On the contrary, the TPB indicates that consumers’ subjective attitude towards the convenience of online takeout, the normative influence of people around them, and their PBC over takeout would promote their purchase intention. For the sake of understanding this consumer trade-off, it is necessary to analyze consumers’ online takeout consumption behavior from the combined perspective of its positive and negative effects (Li and Wang, 2022). Therefore, we aimed to fill the second research gap by integrating the CPR and the TPB and considering the differences in consumers’ education levels to explore the mechanism through which the perceived pollution risk of takeout packaging affects consumers’ takeout consumption.

By addressing the abovementioned two gaps, the theoretical contributions of the present study are threefold. First, considering the new problems arising from online takeout, this study expands the theoretical propositions of risk perception to the online takeout industry, especially in view of environmental and human health risks as well as their negative impacts on consumer purchase intention. Second, we extend the TPB to the online takeout context, which sheds further theoretical light by considering technological as well as environmental impacts on a traditionally physical industry. Third, the study deepens the relationship between the TPB and perceived risk, thereby improving our understanding of the intermediate (i.e., attitude, subjective norms, and PBC) and moderating mechanisms (i.e., consumers’ education level) of how the perceived risk of packaging pollution negatively affects online takeout purchase intention. For marketing practitioners, the results of our study provide insights that can boost their performance. Specifically, we explain consumers’ risk perception factors of packaging pollution from online takeout, which helps online food retailers improve their packaging strategy. We also show how perceived packaging pollution risk affects consumer purchase intention through the TPB and what is the discrepancy among different consumers’ education levels, thereby laying a foundation for the long-term prosperity of the online takeout industry and sustainable consumption.

## Literature review

### Concept of perceived risk (CPR)

When consumers choose an online platform as a shopping channel, they are able to enjoy a wealth of choices, more detailed product descriptions, and comparable prices (Schuermann and Woo, 2022). However, due to the intangible nature of online transactions, consumers may also need to bear some unpleasant consequences caused by unexpected product performance, payment issues, and problems in the delivery process—this is known as perceived risk. The term ‘perceived risk’ was first widely used in the field of psychology and is an important individual psychological perception factor affecting the consumer purchase process (Mitchell, 1999). Since Bauer (1960) extended the concept of perceived risk from psychology to consumer behavior, many scholars have researched and proposed different explanations of perceived risk. Cox (1967) and Cunningham (1967) summarized perceived risk into two core elements, i.e., uncertainty before the purchase and the severity of consequences, which have been widely accepted and used in subsequent studies. Slovic and Peters (2006) referred to perceived risk as “the affect heuristic”, which is closely related to developing regulation and public policy for understanding and evaluating the perceived risk (Slovic, 2011) and analyzed the gap between expert view of risk and public perceptions (Slovic, 2016).

Concerning the perceived risk of online takeout packaging, it refers to a series of possible uncertain situations that are subjectively inferred by consumers about takeout packaging (Schuermann and Woo, 2022). Thus, perceived risk differs from objective risk in that it exists only due to the subjective judgment of consumers. It is not necessarily real, so it has a certain degree of indeterminacy. It also includes two perspectives. On the one hand, it is the perception of loss that may be caused by takeout packaging before the purchase; on the other, it is the perception of how severe the consequences of this loss would be after the purchase.

In terms of its composition, Cox (1967) pointed out that perceived risk includes two aspects: financial risk and psychological risk. Based on these early findings, other scholars have conducted more in-depth research on the dimensional composition of perceived risk. Notably, Jacoby and Kaplan (1972) synthesized the risk literature of numerous instances to summarize five consumers’ perceived risks: physical risks, financial risks, social risks, functional risks, and psychological risks and investigated their relationships (Kaplan et al., 1974). As we enter the era of the Internet economy, consumers’ perceived risk varies in shopping mode, content, and dimensions, especially concerning the delivery of food that is not easily stored and is supposed to consume instantly when food products are produced (Quevedo-Silva et al., 2016; Cai and Leung, 2020). Pillai et al. (2022) emphasized three perceived risks, i.e., psychological risk, financial risk, and product risk which influenced consumers’ using intention of online food services. While in the context of drone food delivery, the risks of time, performance, and privacy are supposed to be a concern (Hwang and Choe, 2019). Apparently, compared to eating in physical restaurants, online takeout services reduce risks to become contaminated by COVID-19 (Zanetta et al., 2021), and the risk dimensions and behaviors that influence overall risk perception vary depending on the risk’s context and its role (Sitkin and Pablo, 1992; Sitkin and Weingart, 1995).

Therefore, it is worth noting that the perceived risk dimension of online shopping is slightly different from that of the traditional environment. In the online shopping environment, consumers have a higher degree of perceived risk pertaining to three aspects: personal finance, privacy, and product authenticity (Liao et al., 2021). However, for online takeout, consumers are already familiar with the operation of online payment; as such, they are less concerned about financial and privacy risks even though they perceive these tools as unsecured (Tinmaz and Doan, 2022). For example, Chinese consumers commonly use payment channels like Alipay and WeChat. Instead, they pay more attention to the risk of product quality, including the impact of takeout on the body as well as the surrounding environment. On the one hand, with the improvement in consumption levels, people are more concerned about the safety of takeout packaging (Xie et al., 2021) and its influence on their health (Schuermann and Woo, 2022). Takeout packaging materials are mainly made of plastic, which releases harmful substances when packed with hot food—this is often reported as a negative side effect. On the other hand, with the accelerating pace of life, consumers have less time to cook their own meals. Online takeout platforms have thus stimulated the demand for takeout while concurrently giving rise to environmental pollution problems (Liu et al., 2020).

### Pollution risk of takeout package

Takeout packaging refers to the packaging of takeout food with the purpose to protect it from external pollution and damage, as well as to maintain its nutritional value and original state during transportation (Schuermann and Woo, 2022). However, for the fact that the food packaged can be contaminated with chemical components through contact and cause potential risks, the safety and pollution of takeout packaging materials become an important indicator to measure food safety and environmental protection (Han et al., 2021; Haque and Fan, 2022). The most widely used takeout food packaging material is plastic (Liu et al., 2020), due to the fact that (i) raw materials are cheap and easy to obtain, and the profit margin is large; (ii) simple processing method can meet the needs of different food packaging; (iii) lightweight and easy to carry; (iv) good performance of gas retention, seepage prevention, and heat sealing; and (v) good chemical stability, acid, and alkali corrosion resistance (Hafsa et al., 2022).

Not only is the widespread use of plastic packaging convenient for human life, but it is also harmful to the environment and human health (Wang and He, 2021). The production, use, and recycling of plastic packaging are accompanied by environmental pollution. There is evidence that many plastic packaging production enterprises fail to meet environmental protection standards, and lots of carbon emissions are generated (Angnunavuri et al., 2022). Additionally, the use of plastic has increased enormously while recycled is very few (Toensmeier, 2020), resulting in pollution of rivers and oceans (Toensmeier, 2020). In natural circumstances, plastic is difficult to degrade and spreads globally with external forces such as rivers, wind, and ocean currents, inevitably leading to environmental contamination (Lebreton et al., 2017).

Consequently, the environmental pollution caused by plastic packaging results in great harm to the organism (Sridharan et al., 2022). Even though plastics, which are mainly and commonly made of microplastics polyethylene (PE), polypropylene (PP), polyethylene glycol terephthalate (PET), etc., are non-toxic, microplastics (MPs) (Liu et al., 2019) and the additives in them, i.e., plasticizer phthalates (PAEs) and bisphenolics (BPs), have varying degrees of toxicity (Sridharan et al., 2022). Under heated circumstances, they are easily transferred to the food in contact with them, and through the diet into the human body and gradually accumulated, resulting in a variety of toxic effects and damage to health (Yang et al., 2019). Some chemicals have also been found to be carcinogenic, teratogenic, and mutagenic (Bonanomi et al., 2022). As MPs are difficult to degrade, once entering the organism, they will cause many mechanical damages to the intestinal system, such as obstruction of dietary organs and the digestive tract, pseudo-satiation that results in the reduction of feeding efficiency, intestinal dysfunction, malnutrition, slow growth, abnormal behavior, injury, and even death (Prata, 2018; Ali et al., 2021). Although MPs can be excreted from the body’s metabolic system, there is still a small amount of residual accumulation in the gut, which can pass through the intestinal wall and cause damage to other body organs. Previous studies have confirmed that nine kinds of MPs with a diameter of 50–500 μm have been detected in human excrement. When the diameter of MPs is <150 μm, they can enter the blood circulation and lymphatic system of the human body through the intestinal tract (Smith et al., 2018). MPs residues have also been detected in many products consumed by consumers, such as drinking water (Semmouri et al., 2022), bivalves, fish, and holothurians (Rios-Fuster et al., 2022). MPs also exist in tea plastic bags frequently used by consumers, thus the maximum exposure of consumers to MPs can reach 1.1 × 10^4^ (Hernandez et al., 2019). The ease of ordering food online has boosted sales of seafood and tea products, while it has also made consumers more vulnerable to MPs.

In terms of plasticizers, studies have shown that shocks, sun exposure, high temperature, microwave heating, and cooking before consumption during the transportation of packaged food can accelerate the migration of plasticizer PAEs and BPs in plastics to food, thus threatening human health. Migration quantity is affected by food type, pH, temperature, and storage time. The longer the exposure time, the greater the migration (Yang et al., 2019). Six harmful ingredients, including Bisphenol A (BPA) which is one of the common BPs, can be released from plastic meal boxes when they are stored in high-temperature soup (>65 °C). As a result, it will cause harm to the human reproductive system after being consumed or drunk (Freire et al. 2006). Even though the toxicity of most plasticizers is low, long-term exposure can still cause significant damage to the human body. Study on PAEs residue in children from various countries has confirmed that plasticizer PAEs keeps accumulating in the body for a long time, leading to human complications (Ringbeck et al., 2022). Besides, PAEs can enter the body through food contact, skin contact, and breathing. After PAEs in takeout food packaging are consumed by the human body, it releases toxic substances that can reduce reversible memory and harm the normal function of the nervous system (Segovia‐mendoza et al., 2022). Notably, BPA can reduce the activity and survival rate of cells, especially causing oxidative damage to proteins, posing a threat to human health (Ďurovcová et al., 2022).

Consequently, the growing number of takeout orders entails the generation of more takeout packaging pollution and brings challenges to environmental governance. Countries around the world have reached a consensus on the toxicity of BPA and implemented clear regulations on the amount of BPA migration in plastics (e.g., China and Korea 0.6 mg/kg, European Union 0.05 mg/kg, Japan 2.5 µg/g) (China Science Testing, 2022).

To sum up, it can be surmised that the perceived risk of online takeout is mainly concentrated on packaging pollution. To highlight the characteristics of takeout, this article focused on the perceived risk of pollution caused by online takeout packaging, including physical harm and environmental pollution. Therefore, we termed this concept packaging pollution risk perception (hereafter PPRP).

### Theory of planned behavior

The TPB prescribes that attitude, subjective norms, and PBC are the main variables that determine behavioral intention. The more positive one’s attitude, subjective norms, and PBC, the greater his/her behavioral intention (Ajzen, 1991; Lim et al., 2022). Attitude is the evaluation of an individual’s likes or dislikes towards performing a specific behavior; subjective norms refer to the social pressure perceived by an individual, reflecting the influence of others on his/her decision-making; and PBC is an individual’s perception of the difficulty of performing a specific behavior, which represents his/her awareness of the behavior that promotes or hinders its performance.

The TPB has been widely supported across various contexts of human behavior research. For example, D’Souza (2022) deploys TPB to explain consumers’ purchase and intention to purchase behavior towards game meats. The three main TPB variables also explain the difference in farmers’ willingness to read and use the risk information on pesticide labels (Bagheri et al., 2021). Similarly, with regard to the voluntary blood donation behavior of higher education students, the TPB variables explain 61.3% of the variance in donation intention (Aschale et al., 2021). In addition, Lim and An (2021) verified the effectiveness of the TPB in explaining Korean consumers’ intention to consume healthy food, and Yang et al. (2022) studied farmers’ intention to adopt low-carbon agricultural technology were positively affected by behavioral attitude, subjective norm and perceived behavioral control based on TPB. Therefore, the TPB provides a reliable framework for studying the influencing factors of online takeout intention. Nonetheless, research needs to combine the antecedents of the TPB with other theories to comprehensively reveal the factors driving individual behavior (Ajzen, 1991; Lim et al., 2022). Taking the perceived risk of online takeout packaging as an antecedent of the TPB, this paper explains the purchase intention of online takeout from the perspective of environmental protection, thereby enriching and supplementing the study of TPB from the perspective of sustainable consumption.

## Theoretical framework and hypotheses

**Fig. 1:**
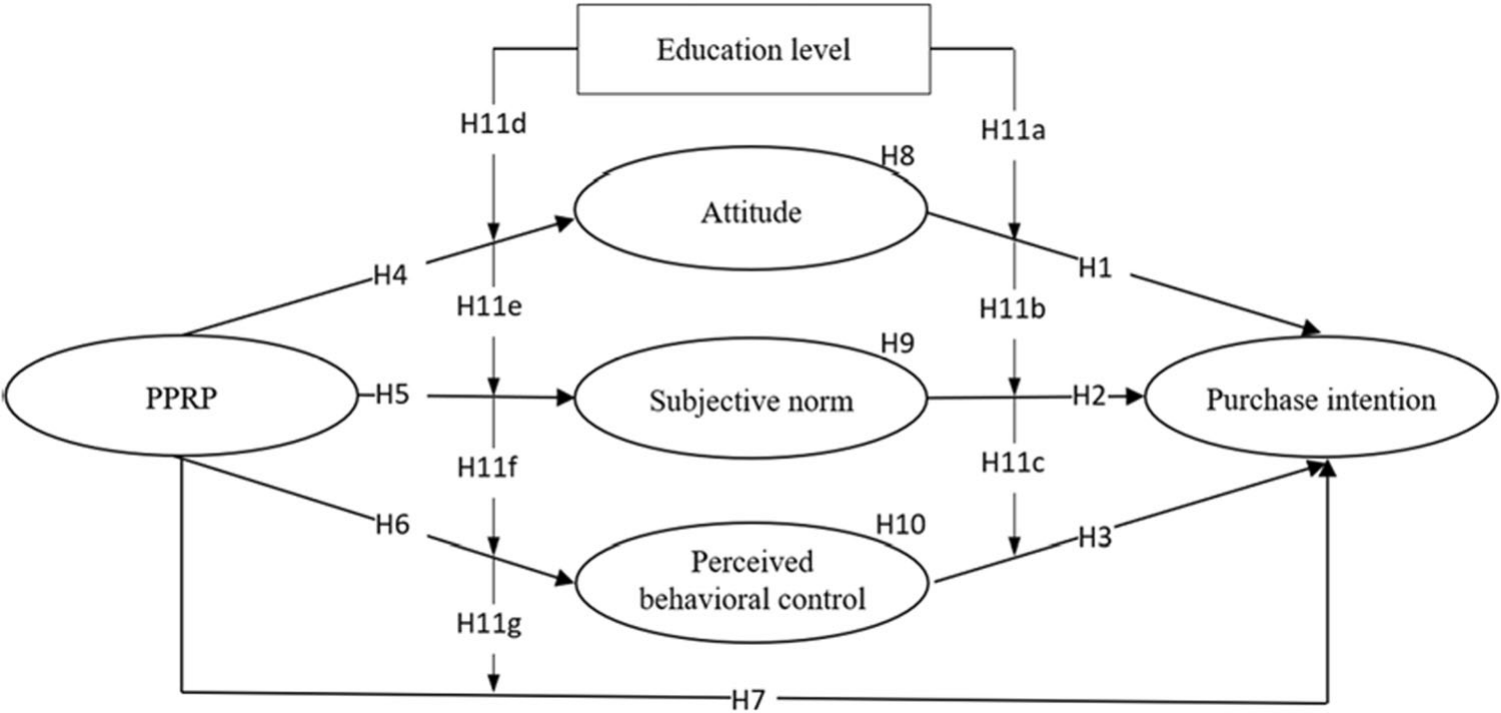
Research model.

TPB provides an approach to understanding what factors facilitate people’s intention to behave in a certain way (Ajzen, 1991), while TPR offers the external causes that hinder people to perform an action in order not to bring about negative effects (Mitchell, 1999). We employed TPB and TPR as theoretical grounds in the current study for three principal reasons. First, they are popular theories that have been well-established to examine the subtleties of people’s behavior intention and actual action (Liao et al., 2021; Lim et al., 2022). Second, the previous studies have demonstrated that these theories are valid in explaining consumers’ behavior intention under some parallel contexts to the current study, such as green hotels (Yeh et al., 2021), healthy food (Lim and An, 2021), package delivery services (German et al., 2022), waste storing behavior (Govindan et al., 2022). Third, the combination of TPB and TPR compromises subjective and objective aspects that shape consumer behavior intention, providing an umbrella underpinning to explore online takeout consumption behavior (Dong and Ge, 2022; Kumari et al., 2022). Therefore, the integration of these theories is beneficial to understanding the influential mechanism between PPRP and consumers’ intention towards ordering online takeout. In accordance with TPR, the specific connotations are concluded under certain contexts while this study based on the extent of literature on packaging pollution defined it as environmental and health risk perception of online takeout packaging. Likewise, the TPB concerning online takeout refers to consumers’ attitude, subjective norm and PBC, and consequently their impacts on the purchase intention of online takeout. Thus, we utilized the TPR and TPB to investigate the specific contextual mechanism of online takeout consumption behavior considering the distinction between consumers’ education levels (see Fig. 1).

### Purchase intention of online takeout based on the TPB

Attitude refers to individuals’ positive or negative evaluations of specific behaviors. To a certain extent, it is manifested as people’s significant belief in the probable outcomes of adopting and implementing specific behaviors. Studies have shown that consumers’ attitude has a significant positive effect on behavioral intention. For example, Lim et al. (2022) found that attitude has a positive impact on the intention to continue using an e-wallet app through an empirical study. Similarly, Leeuw et al. (2015) analyzed the environmental behavior of young people through the TPB and verified that attitude positively impacts their environmental intentions. In the present context, consumers’ attitude towards online takeout is embodied in their cognitive beliefs about takeout products’ speed and convenience, which are shaped by the Internet. Because online takeout purchase can be almost immediately delivered to consumers by designated merchants, consumers feel that online takeout purchase is fast (Guo et al., 2022). In addition, consumers can place orders for online takeout on their mobile phones without having to leave their homes and buy in person. This makes online takeout purchases convenient to consumers, which has positive implications for their purchase intention (German et al., 2022). Subjective norms refer to the external normative forces that influence individuals to undertake a particular behavior. When they are consistent with attitude, they have a stronger role in promoting behavioral intention. Numerous research results show that subjective norms positively affect people’s behavioral intentions on different occasions, such as farmers’ intention to adopt low-carbon agricultural technology in China (Yang et al., 2022), and consumers’ intention to buy healthy food (Lim and An, 2021). As far as this research is concerned, consumers are often influenced by the people around them who recommend them to buy takeout. Thus, the positive guidance of subjective norms towards takeout may enhance consumers’ purchase intention.

The PBC component of the TPB represents the expansion and improvement of the Theory of Reasoned Action (TRA). Like other components of the TPB, PBC has been shown to influence purchase intentions. For instance, Paul et al. (2016) confirmed the influence of PBC on green product purchase intentions. In the current study, PBC mainly refers to the customers’ perceived difficulty in the process of using online takeout platforms. With the rapid development of the Internet, the functions of various takeout applications are constantly being optimized with better usability (Davis, 1989; Hakim et al., 2022), making it more and more convenient for consumers to buy online takeout. Therefore, consumers can now save time and easily buy takeout with a strong PBC, which promotes purchase intention.

Therefore, from the online takeout perspective of TPB, ordering online and eating offline is so convenient and fast that consumers develop a positive attitude towards it. In addition, purchasing behavior is influenced by the recommendations of people around consumers, and they themselves recommend takeout to others. Indubitably, this is closely related to the development of online platforms for food delivery and popular mobile smart devices in the hands of consumers, making ordering food delivery online very simple and easy.

Based on the above discussion, we proposed the following hypotheses:

**H1**. Consumers’ attitude toward online takeout positively affects purchase intention.

**H2**. Consumers’ subjective norms towards online takeout positively affect purchase intention.

**H3**. Consumers’ PBC over online takeout positively affects purchase intention.

### The perceived pollution risk of online takeout packaging and TPB

The search for safety is the instinctive self-protection process instilled in humans from birth to development and adulthood. Research by Wu et al. (2020) shows that consumers have perceived risks in online shopping, which negatively influence consumer satisfaction and the perceived usefulness of e-stores. Online takeout customers, in particular, are concerned about the perceived usefulness of online ordering platforms and the quality of the products provided by takeout platforms, which are perceived risks often referred to that affect online consumers’ satisfaction when shopping online. For instance, Faqih (2022) found a negative correlation between perceived risk and consumers’ online shopping behavior. Similarly, Tyrväinen and Karjaluoto (2022) deployed a meta-analysis technique studying 20,538 respondents and then concluded that perceived risks significantly impact consumers’ online grocery purchase intention with attitude as a moderator. Though extant research has shown that perceived convenience, effectiveness, and risks all have significant impacts on consumers’ online purchase intentions, one significant factor that should not be neglected is perceived risk from the outer environment (Afshan and Sharif, 2016; Barnes, 2019; Guo et al., 2021a, 2021b). In fact, it is likely that when consumers understand that the packaging of online takeout may be harmful to their health and the environment, their attitude, subjective norms, and PBC pertaining to takeout will significantly reduce as well. In other words, if consumers deeply perceive that the packaging is hazardous to them and pollutes the environment, consumers will choose to protect the environment and maintain their health, adopting green consumption behavior (Kumari et al., 2022). Consequently, the notion would result in a negative evaluation of takeout behavior and a weaker attitude toward online takeout. Furthermore, to some extent, consumers may question or even oppose the people around them who favor or recommend online takeout (Mucinhato et al., 2022). As a result, the PPRP of takeout is likely to have a negative impact on their subjective norms. Ultimately, if the packaging pollution of online takeout is harmful to the environment and health, online takeout advantages such as convenience, speed, and cost will carry less value (Koch et al., 2022); rather, negative emotions such as anxiety and worry may arise and thereby reduce consumers’ PBC. Ultimately, under these conditions, consumers’ purchase intention of online takeout would decrease.

In line with the above analysis, we proposed the following hypotheses:

**H4**. Consumers’ PPRP of online takeout negatively affects attitudes.

**H5**. Consumers’ PPRP of online takeout negatively affects subjective norms.

**H6**. Consumers’ PPRP of online takeout negatively affects PBC.

**H7**. Consumers’ PPRP of online takeout negatively affects purchase intention.

### The mediating role of attitude, subjective norm, and PBC

With respect to H1–H3, we logically deduced the positive influences of attitude, subjective norms, and PBC on the purchase intention of online takeout. Simultaneously, we predicted that these three factors are negatively affected by PPRP, as proposed in H4–H6. Therefore, it can be inferred that attitude, subjective norms, and PBC have mediating effects on the PPRP–intention relationship. The mediating role of attitude, subject norm, and PBC have been confirmed by some previous studies in different contexts. For instance, Yeh et al. (2021) verified that attitude, subject norm, and PBC play the role of mediators between the relationship of beliefs and behavioral intention toward green hotel consumption. According to Wu and Kuang (2021), the mediating effect of attitude and subject norm are examined on the association between status seeking, social interaction, the norm of reciprocity, and the intention to share health information via WeChat. Liu et al. (2022) also claimed that attitude mediated the link between perceived space adequacy, perceived usefulness, perceived ease of use, and behavioral intention toward health information technology acceptance. Furthermore, attitude towards the use of the SANAD App mediated the impact of antecedents that influence the APP adoption on behavioral intention (AlHadid et al., 2022). In this study’s context, PPRP gives consumers a clear understanding of the harmful outcomes of purchase takeout, which may diminish its perceived advantages. Subsequently, this would lead to consumers’ unfavorable attitudes toward online takeout, resulting in their weakened purchase intention (Wu et al., 2020; Faqih, 2022). In addition, PPRP makes consumers hesitate and question the approval and recommendation of the people around them, especially when consumers are aware of the environmental pollution and health hazards from takeout packaging; this inevitably has a negative effect on their purchase intention (Mucinhato et al., 2022; Koch et al., 2022). Finally, consumers’ higher perception of takeout packaging pollution risk will nullify the advantages of convenience and ease of buying takeout through the Internet (Paul et al., 2016). Instead, consumers will focus on the importance of the environment and their own health, thereby minimizing their PBC over online takeout and resultantly, decreasing their purchase intention. In short, PPRP may negatively affect purchase intention by weakening consumers’ attitudes, subjective norms, and PBC.

Therefore, we proposed the following hypotheses:

**H8**. Consumers’ attitude toward online takeout mediates the relationship between PPRP and purchase intention.

**H9**. Consumers’ subjective norms towards online takeout mediate the relationship between PPRP and purchase intention.

**H10**. Consumers’ PBC over online takeout mediates the relationship between PPRP and purchase intention.

### The moderating effect of educational level

While the concept of perceived risk has established the associations of certain risk factors with people’s behavior intention, some research revealed these relationships to be inconsistent due to the presence of such personal or circumstantial factors as suggested by Herrmann et al. (2022). Education is one such personal contributor that previous research has demonstrated to impact purchase intention (Li et al., 2022), particularly in the scenarios of food choice (Marsola et al., 2020), food consumption (Hristov et al., 2022), and food-related products (Martins et al., 2022). People with higher education levels are more inclined to put a premium on their health, thus buying less takeaways (Janssen et al., 2018). While Zhang et al. (2022), considering the interaction of education level and environmental factors (e.g., air quality and weather conditions), pointed out that higher educated people are prone to consume more takeout food, especially in developed areas or cities (Li et al., 2022). This elucidates that the relationship between education level and purchase intention cannot be investigated separately from both external objective factors (e.g., perceived risks of takeout packaging) and internal subjective motivations (e.g., attitude, subjective norms, and PBC) but should be integrated into a comprehensive way to capture a better understanding of the mechanism of education level. Therefore, education level is crucial to appropriately weighing the risk probability and its outcomes, because if the consumers attained higher education, the more wisely their culturally acceptable behaviors are displayed (Oribe-Garcia et al., 2015; Han et al., 2018).

Likewise, for accounting for the inconsistency of the extended TPB model, the moderating effect between initial TPB variables and external drivers is supposed to be considered, as suggested by Conner (2015). In other words, the extent to which the validation of various TPB extensions has reached across a wide variety of behaviors and demographics remains to be examined. To this end, the significance of education level as a mediator in TPB was highlighted in prior studies. For instance, in the scenario of safe food handling, consumers with higher education levels are less affected by PBC than their counterparts (Ruby et al., 2019). Meanwhile, in terms of electric vehicles, the impact of PBC on purchase intention towards electric vehicles is stronger for the higher educated consumers than the less ones (Huang et al., 2022). Concurrently, the education level moderated the association between attitude and purchase intention, wherein the impact path is strengthened among consumers with higher levels of education. Furthermore, the impacts of education level on popular attitudes and actions have already been proven to be larger, whilst the effects on ethnic beliefs and behaviors have been examined to be smaller with rising education level (Yagmur and van de Vijver, 2012). In the context of online takeout, with the prevailing attention to the environment and human health under COVID-19, the effects of people’s education level on their relative subjective psychological judgment, i.e., perceived risk of takeout packaging, attitude, subjective norms, PBC, and purchase intention, are still worth exploring.

Therefore, it is necessary to evaluate in this study how education level may function as a moderator of the effects of PPRP, attitude, subjective norms, and PBC on purchase intention towards online takeout. Little study has thoroughly examined this integrated model in the context of online takeout behavior, despite the possibility that the factors discussed, such as attitude, social norms, and PBC along with education level, may also affect online takeout purchase behavior. According to Talwar et al. (2020a), research can provide light on the subtle variations in customer behavior by looking at moderating variables. In order to explore the granular nuances of the relationships in this study, we propose that education levels moderate the associations between PPRP, attitude, subjective norms, and PBC in the context of online takeout. Our supposition is in line with other research that claimed education level might operate as a moderator (Janssen et al., 2018; Li et al., 2022). Consequently, this leads to the hypothesis as followed:

**H11.** (**a–g**) Education level moderates the relationships between (**a**) attitude and purchase intention, (**b**) subjective norms and purchase intention, (**c**) PBC and purchase intention, (**d**) PPRP and attitude, (**e**) PPRP and subjective norms, (**f**) PPRP and PBC and (**g**) PPRP and purchase intention.

## Methodology

### Measures

The measurement items pertaining to TPB used in the research questionnaire were mainly derived from previous studies (Ajzen, 1991; Aschale et al., 2021; Yang et al., 2022) and were modified to suit the research context of online takeout (see Table 1). In terms of perceived risk, many researchers employed different measurements grounded on various scenarios which mainly adapted from previous studies. For instance, Hwang and Choe (2019) used 15 items (3 items per construct) to measure the perceived risk of drone food delivery, while Pillai et al. (2022) employed 7 items to measure online food delivery risks. However, there is little research that directly measures consumers’ perceived risk of packaging pollution in the context of online takeout (Shankar et al., 2022). In order to operationalize the concept of PPRP, we carry out an in-depth interview of 20 Chinese online takeout consumers through purposive sampling to provide insight into specific online takeout packaging pollution risk perception: PPRP. Based on the concept of perceived risk (Cox, 1967; Cunningham, 1967), the PPRP refers to the uncertain the package may bring about on the environment and human health, as well as the severity of such repercussions, which comes from the pollution generated by the process of producing, using, and recycling packaging. The approach to content analysis of the interview material is based on the grounded theory—a qualitative method, which is precious when objective phenomena need exploring and are not explained enough (Gawlik, 2016). Grounded theory is beneficial either to form an emerging theory (Strauss and Corbin, 1994) or develop new concepts through systematical coding (Tandon et al., 2021). For example, Traynor et al. (2022) used grounded theory investigating the emergency of third-party online food delivery and Wang et al. (2022) explored the factors influencing consumers’ food safety self-protection behavior based on grounded theory, while Tandon et al. (2021) employed grounded theory developing the measurements of delivery food consumption value. Thus, this study utilized grounded theory to conduct the content analysis and extract measurement instrumentation of PPRP pertaining to online takeout.

**Table 1:** Reliability test results of the pilot survey.

Construct	Item	Correlation^a^	Cronbach’s alpha^a^	Cronbach’s alpha
PPRP	PPRP1: Takeout packaging can cause environmental pollution	0.724	0.821	0.862
	PPRP2: Takeout packaging will affect the ecological balance	0.769	0.800	
	PPRP3: Takeout packaging recycling is not proper, resulting in pollution	0.699	0.827	
	PPRP4: Online takeout packaging may be harmful to humans	0.671	0.848	
AT	AT1: The contactless delivery of online takeout is safe	0.593	0.608	0.735
	AT2: The smart delivery of online takeout is fast	0.553	0.657	
	AT3: Online takeout is convenient for reducing traveling	0.533	0.680	
SN	SN1: Users of the social media platforms I use recommend online takeout	0.515	0.780	0.790
	SN2: My favorite network influencers recommend online takeout	0.673	0.700	
	SN3: People around me understand me ordering takeout online	0.673	0.700	
	SN4: People around me recommend me to order takeout online	0.543	0.766	
PBC	PBC1: I can use mobile phone to order online takeout easily	0.530	0.662	0726
	PBC2: I can buy takeout online for less money than offline	0.568	0.621	
	PBC3: I can cancel or change my online takeout order easily	0.551	0.636	
PI	PI1: I will often buy online takeout food	0.681	0.677	0.796
	PI2: I would recommend online takeout to people around me	0.581	0.782	
	PI3: I would preferentially use online platforms to order takeout	0.666	0.701	

*AT* attitude, *SN* subjective norms, *PBC* perceived behavioral control, *PPRP* packaging pollution risk perception, *PI* purchase intention.

Correlation^a^ means correlation between revised item and total score.

Cronbach’s alpha^a^ means cronbach’s alpha after deleting the item.

The interviewees had ordered online takeout at least once a week for three months before the interviews. Each interview lasted about 15–30 min by two researchers taking notes. The participants were asked about the positive aspects of online takeout, as well as the negative impacts of its packaging, whereby they were able to comment on the online takeout genuinely to avoid bias (Tandon et al., 2021).

The results of the interviews revealed that PPRP was mainly derived from concerns about the environment (e.g., “the materials of takeout are not environmentally friendly”, “The takeout packaging is difficult to degrade and pollutes the environment”, and “the recycling process of packaging waste indirectly pollutes the environment”) and human health (e.g., “plasticizers and other chemicals in takeout packaging are health hazards”), which were in line with the previous studies about packaging risks (Toensmeier, 2020; Wang and He, 2021; Angnunavuri et al., 2022). To delete the irrelevant items and combine the similar ones, there are six items concerning PPRP left to form the initial measurements. Furthermore, three professors were invited to review the items pertaining to their relevance and face validity. The panel advised deleting two items and recommended minor rectifications of the left four items. To include parsimonious items is beneficial to improve model fit (Hair et al., 2018). The number of items for a construct in this study satisfies the analytical requirement as suggested by Kenny (1979), who proposed the rule of thumb for indicators’ number: “Two might be fine, three is better, four is best, and anything more is gravy”, which was supported by other scholars (Bollen, 1989; Kline, 1998; Mulaik, 1994; Hinkin et al., 1997). Equally, Noar (2003) held that four items were enough to frame an effective construct. Finally, the final pool of items in this study was checked again by the panel (see Table 1). This procedure was in alignment with the protocols suggested by Saunders et al. (2019), which have adopted a parallel approach to extract context-specific measurements (e.g., Tandon et al., 2021).

All items were measured on a 5-point Likert scale, from “strongly disagree” to “strongly agree”. In addition, the original English items were first translated into Chinese and then translated back into English for comparison with the original (Brislin, 1970). The translated items were repeatedly checked by two marketing scholars and two entrepreneurs for accuracy. The opinions of a number of consumers were also solicited to ensure the translation validity and content validity of the questionnaire items.

A pilot investigation in China was conducted to gauge respondents’ preliminary understanding of the items and avoid low data credibility due to ambiguity in the formal investigation stage. A total of 116 samples participated in the pilot study to confirm the reliability (Nunnally and Bernstein, 1994) (see Table 1) and validity (Tabachnick and Fidell, 2007) (see Table 2) of the questionnaire. After the pilot survey, a formal survey (*n* = 336) was conducted to further verify the questionnaire’s reliability and validity (see Table 4), and subsequently to test the hypotheses of the research model using SPSS and AMOS software. The sample size of 336 is up to the standard minimum sample size of 100 required to perform the hypotheses test via structural equation modeling (Hair et al., 2018), as there are five constructs with more than three items, and with high item communalities (higher than 0.6) in the research model.

**Table 2:** Validity test results of the pilot survey.

Construct	Item	Component factor loading					Communalities
		1	2	3	4	5	
PPRP	PPRP1	0.819	−0.126	−0.192	−0.109	−0.066	0.74
	PPRP2	0.805	−0.184	−0.086	−0.194	−0.247	0.788
	PPRP3	0.779	−0.173	−0.111	−0.148	−0.163	0.697
	PPRP4	0.742	−0.096	−0.080	−0.348	−0.056	0.691
SN	SN3	−0.081	0.745	0.175	0.154	0.059	0.766
	SN1	−0.154	0.738	0.133	0.161	0.156	0.72
	SN2	−0.171	0.730	0.118	0.179	0.097	0.621
	SN4	−0.154	0.721	0.308	0.093	0.187	0.637
AT	AT1	−0.058	0.239	0.805	0.166	0.171	0.618
	AT2	−0.107	0.222	0.746	0.273	0.169	0.619
	AT3	−0.285	0.201	0.685	0.005	0.175	0.682
PI	PI1	−0.177	0.192	0.183	0.810	0.130	0.666
	PI3	−0.333	0.197	0.250	0.715	0.093	0.678
	PI2	−0.281	0.255	0.023	0.667	0.228	0.703
PBC	PBC3	−0.185	0.285	0.022	0.106	0.759	0.776
	PBC2	−0.145	0.145	0.307	0.026	0.0736	0.641
	PBC1	−0.112	0.018	0.217	0.299	0.719	0.733
Eigenvalue		6.743	1.738	1.257	1.025	1.013	–
Explain the total variance%		17.32	15.489	12.535	12.355	11.573	–
Cumulative explained variance%		17.32	32.809	45.344	57.698	69.271	–

*AT* attitude, *SN* subjective norms, *PBC* perceived behavioral control, *PPRP* packaging pollution risk perception, *PI* purchase intention.

### Data collection

While consumers of online takeout are widely distributed across China, they are mainly comprised of young consumers in large cities (China Industrial Research Institute, 2022). Data collection for the pilot study and actual study was conducted through the online survey platform WJX.cn. Online data collection is not only quick in retrieving data from young consumer groups but is also convenient for accessing regionally dispersed consumer data efficiently at a low cost (Dirsehan and Cankat, 2021; Akram et al., 2020). Furthermore, we employ quota sampling combined with a purposive sampling technique to select the respondents for the achievement of the research goals, as in lots of research in the online retail context (Cheah et al., 2022; Lim and An, 2021).

Specifically, to avoid measurement error caused by regional differences, the sample respondents of this study were selected from popular cities in different Chinese regions, namely Beijing (18%), Shanghai (16.5%), Guangzhou (17%), Chengdu (16.8%), Wuhan (15%), and Xi’an (16.7%). The number of respondents in each city was distributed equally by sex to represent the typical characteristics of Chinese online takeout consumers. An important criterion for the sample was that the consumers surveyed had to have prior experience in buying takeout through the Internet. To ensure that their answers reflected their purchase experience as much as possible, the first part of the questionnaire included the filter question “Have you bought takeout on the Internet in the last week?” Only consumers who answered ‘yes’ were allowed to continue with the questions. The second part of the questionnaire presented the measurement items of the research variables, while the last section solicited respondents’ demographic information, such as sex, age, occupation, and educational background, which serve as control variables to prevent deviation of hypotheses test resulting in spurious explanation (Wang et al., 2022).

To avoid common method bias (CMB) in the study, prior procedural controls (Fuller et al., 2016) and post-statistical tests were implemented (Podsakoff et al., 2003, 2012). Procedure controls were designed directly for the source of CMB, i.e., the respondents. First, the items of the questionnaire were adopted from previous research and were as short and clear as possible. Second, scholars and practitioners in the field of consumer behavior were asked to review the items. They verified that there were no obscure and difficult terms in the items and that the descriptions of the concepts were unambiguous. Finally, we protected the anonymity of the respondents and informed them before the investigation that there were no right or wrong answers. This was to prevent respondents from guessing ahead or answering in socially desirable manners, as per the implicit theory effect.

To supplement these procedural measures, the collected data were statistically tested for CMB. The method adopted for this purpose was confirmatory factor analysis (CFA) (Podsakoff et al., 2012), as Fuller et al. (2016) suggested that Harman’s single factor test may generate inaccurate conclusions about CMB and an informed choice should be made concerning post-hoc approaches to address CMB. Therefore, the five-factor confirmatory model established by the five latent variables involved in this study was compared with the single-factor confirmatory model of one latent variable composed of all test items. A significant difference implies that CMB is well-controlled. The five-factor confirmatory model of the pilot survey data (*χ*^2^/DF = 1.090, GFI = 0.891, AGFI = 0.847, CFI = 0.987, NFI = 0.871, IFI = 0.988, RMSEA = 0.028, SRMR = 0.0497) fit better than the single factor model (*χ*^2^//DF = 2.616, GFI = 0.720, AGFI = 0.640, CFI = 0.755, NFI = 0.661, IFI = 0.760, RMSEA = 0.119, SRMR = 0.092), with a significant difference in Chi-square values between them (Δ*χ*^2^/ = 192.409, Δdf = 10). Similarly, in the actual research, the model fit of the five-factor model (*χ*^2^/DF = 1.067, GFI = 0.962, AGFI = 0.947, CFI = 0.997, NFI = 0.952, IFI = 0.997, RMSEA = 0.014, SRMR = 0.030) was better than that of the single factor model (*χ*^2^/DF = 7.681, GFI = 0.716, AGFI = 0.634, CFI = 0.649, NFI = 0.620, IFI = 0.652, RMSEA = 0.141, SRMR = 0.1024), and there was a significant difference in Chi-square values between them (Δ*χ*^2^ = 696.282, Δdf = 7). Hence, any potential CMB in this study was well-controlled.

## Data analysis and results

This study employed confirmatory factor analysis (CFA) to verify the reliability and validity, structural equation analysis to test the direct (H1–H7) and indirect (H8–H10) effects of latent variables in the proposed model, and multi-group structural equation analysis to examine the moderating effect (H11a–H11g) of education level.

### Confirmatory factor analysis

CFA with a maximum likelihood estimation approach was used to examine the reliability and validity of the data via model fitting, composite reliability (CR), factor loadings, and average variance extracted (AVE) (Byrne, 2004; Chin et al., 2008; Byrne, 2009). A total of 336 valid responses were collected in this study, of which 169 were from male consumers (50.3%) and 167 were from female consumers (49.7%) (Table 3). The sample’s demographic distribution reflected the characteristics of takeout consumers as outlined in the 2022 Report on China’s Food Delivery Industry (China Industrial Research Institute, 2022).

**Table 3:** Demographic profile.

Variable	Category	Frequency	Percentage
Sex	Male	169	50.3
	Female	167	49.7
Age	Under 20	65	19.3
	20–30	101	30.1
	30–40	87	25.9
	Over 40	83	24.7
Occupation	Company employee	131	39.0
	Civil servant	32	9.5
	Farmer	10	3.0
	Student	69	20.5
	Other	94	28.0
Education background	Junior high school and below	31	9.2
	Senior high school and technical secondary school	83	24.7
	Junior college	101	30.1
	Bachelor’s degree and above	121	36.0

The fitness index (*χ*^2^/df = 1.067, GFI = 0.962, AGFI = 0.947, CFI = 0.997, NFI = 0.952, IFI = 0.997, RMSEA = 0.014, SRMR = 0.030) indicated that the model fit well (Marsh et al., 1988; Bentler and Bonett, 1980). In addition, all the items had factor loading values above 0.6 (*p* < 0.05) (f), CR values >0.70, and AVE values higher than 0.50, proving that the data achieved the statistical standards of composite reliability and convergence validity ((Bagozzi and Yi, 1988; Kline, 1998; Hair et al., 2018) (Table 4).

**Table 4:** Composite reliability and convergence validity.

Item		Construct	Estimate	SE	CR	Standardized estimate	CR	AVE
PPRP1	←	PPRP	1.000			0.673	0.84	0.571
PPRP2	←	PPRP	1.324^***^	0.104	12.751	0.845		
PPRP3	←	PPRP	1.344^***^	0.107	12.547	0.820		
PPRP4	←	PPRP	1.200^***^	0.113	10.626	0.665		
AT1	←	AT	1.000			0.781	0.792	0.562
AT2	←	AT	1.102^***^	0.086	12.803	0.801		
AT3	←	AT	0.845^***^	0.076	11.113	0.659		
SN1	←	SN	1.000			0.708	0.816	0.526
SN2	←	SN	0.999^***^	0.088	11.313	0.706		
SN3	←	SN	1.017^***^	0.090	11.294	0.705		
SN4	←	SN	1.118^***^	0.091	12.228	0.779		
PBC1	←	PBC	1.000			0.707	0.778	0.54
PBC2	←	PBC	0.975^***^	0.087	11.187	0.774		
PBC3	←	PBC	1.010^***^	0.093	10.818	0.721		
PI1	←	PI	1.000			0.785	0.817	0.598
PI2	←	PI	0.980^***^	0.075	13.076	0.748		
PI3	←	PI	0.924^***^	0.068	13.625	0.786		

*AT* attitude, *SN* subjective norms, *PBC* perceived behavioral control, *PPRP* packaging pollution risk perception, *PI* purchase intention, *CR* composite reliability, *AVE* average variance extracted, *SE* standard error, *CR* critical ratios for difference.

***p < 0.001.

Furthermore, we can see that the square root of the AVE value of each variable was greater than the correlation coefficient of the variable with other variables (Table 5). This shows that the discriminant validity of the data was well-established (Fornell and Larker, 1981). Meanwhile, the correlation coefficients between two latent variables were <0.7, indicating there existed no multicollinearity problem (Grewal et al., 2004). Therefore, the measurement model of this study was validated through the CFA, and subsequently, the structural model was examined to test the hypotheses (Byrne, 2009).

**Table 5:** Discriminant validity.

	PPRP	AT	SN	PBC	PI
PPRP	**0.756**	−0.303^**^	−0.333^**^	−0.342^**^	−0.458^**^
AT	−0.356^***^	**0.725**	0.466	0.383^**^	0.471^**^
SN	−0.392^***^	0.577^***^	**0.773**	0.421	0.478^**^
PBC	−0.421^***^	0.483^***^	0.524^***^	**0.735**	0.439^**^
PI	−0.533^***^	0.582^***^	0.580^***^	0.541^***^	**0.773**

The diagonal is the square root of the AVE value of each variable; above the diagonal is the latent variable’s mean correlation coefficient, and below the diagonal is the latent variable’s correlation coefficient.

*AT* attitude, *SN* aubjective norms, *PBC* perceived behavioral control, *PPRP* packaging pollution risk perception, *PI* purchase intention.

**P <0.01, ***P <0.001.

### Structural equation analysis

**Fig. 2:**
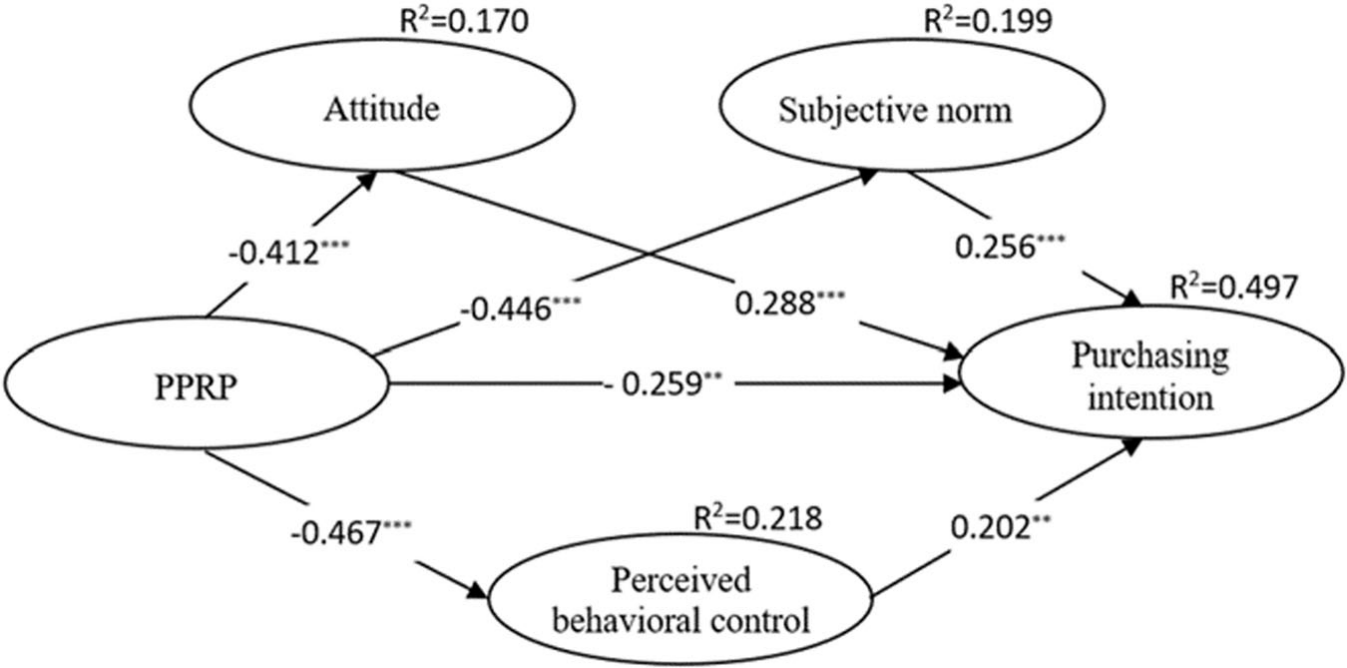
Results of hypothesis test by structural equation modeling. Note: ***p < 0.001, **p < 0.01; path coefficients are standardized values.

The hypotheses are tested by structural equation modeling, whose advantage is that it can evaluate the relationships among multiple independent and dependent variables at the same time (Hoyle, 1995; Byrne, 2009). Moreover, the model can not only uncover the direct effects of exogenous variables (independent variables) on endogenous variables (dependent variables) but also test their indirect influences. The structural equation model was developed using AMOS24.0 software with the maximum likelihood method as the model fitting method (Byrne, 2004). The results (see Fig. 2) revealed that the fitting indexes ((*χ*^2^/df = 1.953, GFI = 0.925, AGFI = 0.897, CFI = 0.953, NFI = 0.909, IFI = 0.953, TLI = 0.943, RMSEA = 0.053) reached the ideal values, indicating that the model fits well (Mulaik et al., 1989; Bentler, 1990; Medonald and Ho, 2002).

The results also showed that the exogenous variables explained almost half of the variance in purchase intention (*R*^2^ = 49.7%), indicating that the combination of the TPB and perceived risk has good explanatory power for the purchase intention of online takeout. In comparison, the TPB antecedents without the external impact of risk perception explained 47.1% of the variance in purchase intention, which is a 2.6% decrease in explanatory power. Meanwhile, the *R*^2^ results reported that the risk perception of online takeout adequately explains the TPB’s influencing factors of takeout purchase intention. Specifically, PPRP accounted for the variance in PBC. According to the path coefficient (*β*) results of the hypothesized relationships, attitude (*β* = 0.288, *p* < 0.001), subjective norms (*β* = 0.256, *p* < 0.001), and PBC (*β* = 0.202, *p* < 0.01) were found to have significant positive impacts on purchase intention, thus supporting H1–H3. Likewise, PPRP demonstrated a significant negative impact on attitude (*β* = −0.412, *p* < 0.001), subjective norms (*β* = −0.446, *p* < 0.001), PBC (*β* = −0.467, *p* < 0.001), and purchase intention (*β* = −0.259, *p* < 0.01). Therefore, H4–H7 were supported.

The mediating effects of attitude, subjective norms, and PBC between the PPRP and purchase intention of online takeout were tested via the bias-corrected bootstrap method in AMOS, specifically with 5000 reiterations at the 95% confidence interval (Zhao et al., 2010). If the confidence interval does not contain a zero, the path is significant. The results (Table 6) indicated that the total effect (TE) [TE = −0.421, 95% CI (−0.519 ~ −0.330)], the direct effect (DE) [DE = −0.186, 95% CI (−0.301 ~ −0.078)], and the indirect effect (IE) [IE = −0.235, 95% CI (−0.338 ~ −0.163)] of PPRP on purchase intention were all significant, indicating that attitude, subjective norms, and PBC partially mediated the relationship. Further bootstrapping analysis of the specific mediation effects of the three variables revealed that attitude [IE_AT_ = 0.085, 95% CI (0.146 ~ 0.045)], subjective norms [IE_SN_ = 0.082, 95% CI (0.139 ~ 0.039)], and PBC [IE_PBC_ = 0.125, 95% CI (−0.338 ~ −0.024)] had significant mediating effects on purchase intention. Among them, the mediating effect of attitude was the largest, accounting for 36.2% of the total indirect effect. The results of PRODCLIN2 analysis (Mackinnon et al., 2007) produced the same conclusion. Therefore, H8–H10 were verified.

**Table 6:** Mediation test results.

Model	Hypothesis	IE_i_	SE	ER	Bias-corrected 95%CI		PRODCLIN2 95%CI		Mediation	Hypothesis supported?
					Lower	Upper	Lower	Upper		
PPRP → AT → PI	H8	−0.085	0.025	36.2%	−0.146	−0.045	−0.153	−0.036	Partial	Yes
PPRP → SN → PI	H9	−0.082	0.025	34.9%	−0.139	−0.039	−0.151	−0.032	Partial	Yes
PPRP → PBC → PI	H10	−0.068	0.026	28.9%	−0.125	−0.024	−0.136	−0.019	Partial	Yes

*AT* attitude, *SN* subjective norms, *PBC* perceived behavioral control, *PPRP* packaging pollution risk perception, *PI* purchase intention. *IE*_*i*_ indirect effects, *i* = AT, SN, PBC, *SE* standard errors; *ER* effect ratio of IE_*i*_ to IE.

### Multi-group structural equation analysis

This study follows the procedures of muti-group analysis (Byrne, 2004). First, this study has divided the sample into three groups, considering the moderating variable of education level measured by nominal scale (Kizgin et al., 2021): (a) respondents of Education Level 1 (*n* = 114) represent education level including senior high school and secondary school and below; (b) Education Level 2 (*n* = 101) with education level of junior college; (c) Education Level 3 (*n* = 121) formed by the education level of bachelor degree and above, with the mean differences and its effect sizes shown in Table 7.

**Table 7:** Mean differences of variables between consumers by education levels.

Variables	Education Level 1		Education Level 2		Education Level 3		Effect size^a^	Effect size^b^	Effect size^c^
	Mean	SD	Mean	SD	Mean	SD			
PPRP	3.482	0.989	3.423	1.012	3.457	1.020	0.433	0.197	−0.243
AT	3.500	0.699	3.469	0.613	3.548	0.686	0.348	−0.534	−0.903
SN	3.450	0.635	3.389	0.632	3.306	0.578	0.704	1.818	1.019
PBC	3.473	0.673	3.544	0.658	3.430	0.665	−0.779	0.500	1.285
PI	3.611	0.723	3.604	0.713	3.606	0.747	0.073	0.053	−0.021

Effect size is defined as the difference in the mean score of Education Level divided by the difference’s standard deviation.

*AT* attitude, *SN* subjective norms, *PBC* perceived behavioral control, *PPRP* packaging pollution risk perception, *PI* purchase intention.

^a^Denotes the effect size between education levels 1 and 2.

^b^Denotes the effect size between education levels 1 and 3.

^c^Denotes the effect size between education levels 2 and 3.

**Fig. 3:**
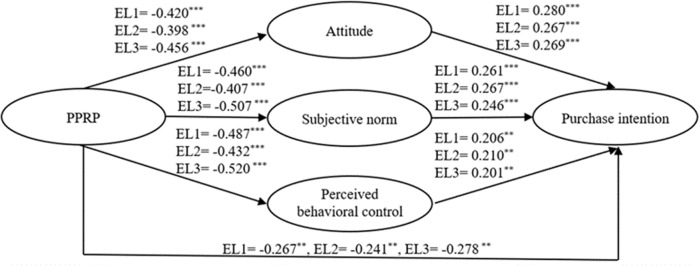
Relationships among latent variables in different education levels. Note: ***p < 0.001, **p < 0.01; EL1 Education Level 1, EL2 Education Level 2, EL3 Education Level 3; path coefficients are standardized values.

Second, an unconstrained muti-group structural model was investigated to measure the configural invariance of the proposed research model in this study. Due to the effect of sample size, the invariance was not supposed to be decided in the light of Chi-square values (Cheung and Rensvold, 2002; Byrne and van de Vijver, 2010). The baseline model across groups showed a good fit (*χ*^2^/df = 1.367, CFI = 0.947, IFI = 0.949, TLI = 0.936, RMSEA = 0.033), given the sample size of 336 (Hair et al., 2018), indicating the factor structure possesses identical characteristic across three education groups. Third, the measurement weights model also suggested that the goodness-of-fit indices (*χ*^2^/df = 1.322, CFI = 0.950, IFI = 0.951, TLI = 0.943, RMSEA = 0.031) met the statistical prerequisite of metric invariance, and its comparison with the unconstrainted model (Δ*χ*^2^ = 16.585, Δdf = 24, *P* = 0.086) indicated parallelly good indices, thus sustaining the measurement invariance (Steenkamp and Baumgartner, 1998). Fourth, the structural weights invariance was examined by the comparison of measurement weights and structural weights model (Δ*χ*^2^ = 57.756, Δdf = 38, *P* = 0.021). Since the results did not support the structural invariance, the partial metric invariance (PMI) was employed wherein the structure path was constrained sequentially to explore in which path the education groups are diverse (Byrne et al., 1989) (see Table 8 and Fig. 3).

**Table 8:** Moderating effect test results.

Model	Hypothesis	*χ*^2^(df) _EL1=EL2_	Δ*χ*^2^	*χ*^2^(df) _EL2=EL3_	Δ*χ*^2^	*χ*^2^(df) _EL1=EL3_	Δ*χ*^2^	Hypothesis supported?
AT → PI	H11a	17.175(25)	0.59	18.693(25)	0.36	18.693	2.108	No
SN → PI	H11b	20.147(25)	3.562	16.731(25)	2.247	16.731	0.146	No
PBC → PI	H11c	19.165(25)	2.58	22.075(25)	0.675	22.075	5.49^*^	Yes
PPRP → AT	H11d	32.791(25)	16.206^***^	16.588(25)	18.631^***^	16.588	0.002	Yes
PPRP → SN	H11e	20.761(25)	4.176^*^	18.864(25)	0.574	18.864	2.279	Yes
PPRP → PBC	H11f	20.903(25)	4.318^*^	16.801(25)	3.086	16.801	0.216	Yes
PPRP → PI	H11g	18.061(25)	1.476	16.75(25)	3.227	16.75	0.165	No

*χ*^2^(df) measurement weights = 16.585(24); *χ*^2^(df) structural weights = 57.756(38).

*EL1* Education Level 1, *EL2* Education Level 2, *EL3* Education Level 3.

***p  < 0.001, *p < 0.05.

**Fig. 4:**
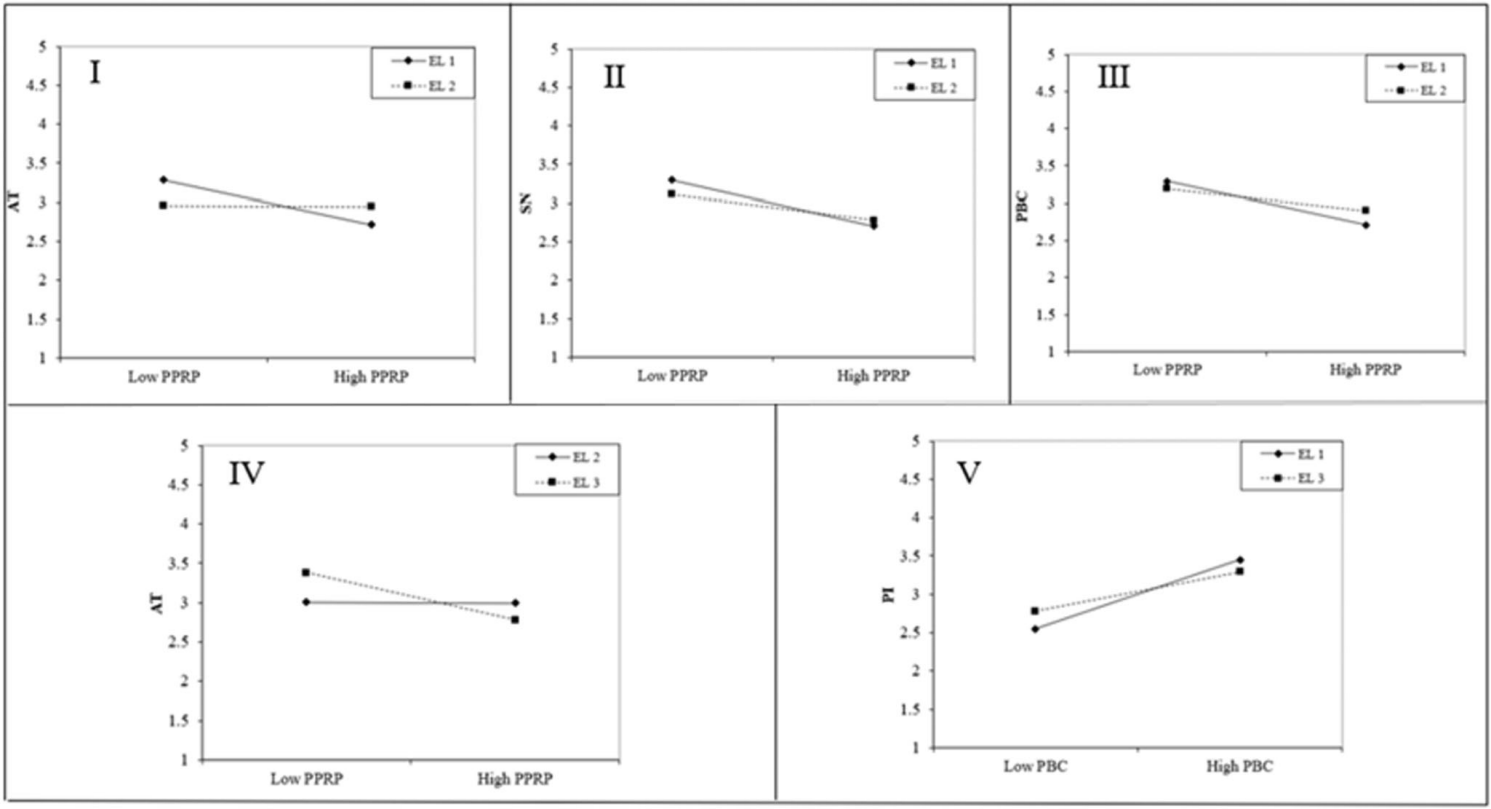
Interaction plots. Note: I denotes moderating effect of Education Level (1 vs. 2) on the association of PPRP and AT; II denotes moderating effect of Education Level (1 vs. 2) on the association of PPRP and SN; III denotes moderating effect of Education Level (1 vs. 2) on the association of PPRP and PBC; IV denotes moderating effect of Education Level (2 vs. 3) on the association of PPRP and AT; V denotes moderating effect of Education Level (1 vs. 3) on the association of PBC and PI.

The results showed that the association between PPRP and AT varies significantly (Δ*χ*^2^ = 32.791, Δdf = 25, *P* < 0.001; Δ*χ*^2^ = 16.588, Δdf = 25, *P* < 0.001) across the EL1 (*β* = −0.420, *P* < 0.001) and EL2 (*β* = −0.398, *P* < 0.001), as well as the EL2 and EL3 (*β* = −0.456, *P* < 0.001) (Fig. 3). The impact of PPRP on SN also differs (Δ*χ*^2^ = 20.761, Δdf = 25, *P* < 0.001) across the EL1 (*β* = −0.460, *P* < 0.001) and EL2 (*β* = −0.407, *P* < 0.001). Both the EL1 (*β* = −0.487, *P* < 0.001) and EL2 (*β* = −0.432, *P* < 0.001) indicated a significant relationship between PPRP and PBC and had significant variation (Δ*χ*^2^ = 20.903, Δdf = 25, *P* < 0.05) across them. Additionally, PBC exerted a positive significant impact on PI for EL1 (*β* = −0.206, *P* < 0.01) and 3 (*β* = −0.201, *P* < 0.01), and the path coefficients were different significantly (Δ*χ*^2^ = 22.075, Δdf = 25, *P* < 0.05). Hence, the H11c, H11d, H11e, and H11f were empirically accepted. The interaction plots of the significant moderating role of EL are given in Fig. 4.

## Discussion and implications

### Discussion of the results

Based on the TPR and the TPB, this study explored how and when the consumers’ perceived risk of online takeout packaging impacted the takeout purchase intention base on the TPR and TPB. The research model was built and tested via structural equation modeling to examine the direct and indirect impact of PPRP on takeout purchase intention through attitude, subjective norms, and PBC, as well as the moderating role of education levels.

This study demonstrates that consumers’ attitude (H1), subject norms (H2), and PBC (H3) of online takeout have a positive impact on purchase intention, whereby attitude has the greatest effect on purchase intention, followed by subjective norms and PBC. After adding PPRP to the model as an exogenous variable, the overall explanatory power of the model improved from 47.1% to 49.7%. The impact of PPRP on TPB is relatively small (2.6%) compared with its influence on attitude (17.0%), subjective norm (19.9%), and PBC (21.8%). This is consistent with the previous conclusions of scholars who verified the TPB in different settings (Lim and An, 2021; Yang et al. 2022). Based on our results, consumers’ attitude towards online takeout is the most important factor determining their purchase intention, which is in line with the previous studies that heightened the role of attitude in TPB, such as Mucinhato et al. (2022) who suggested that attitude to food safety had the greatest impact on its practices. This is because, with the development of mobile networks, consumers can search for their desired takeout and pay online through their mobile phone, which is far more efficient than the traditional offline experience (Li and Wang, 2022), saving consumers’ time and energy by avoiding congestion, queuing, and waiting. Also, in the post-COVID-19 period, online takeout is regarded as an important means for catering businesses to enhance competitiveness (Meena and Kumar, 2022). Taken together, these advantages underscore the strong influence of a positive attitude towards online takeout. In addition, a growing number of consumers are accustomed to ordering online takeout (Xie et al., 2021), which exerts a positive impact on their surrounding people like relatives and friends, i.e., subject norms. The current study sustains the argument of early researchers who proposed the significance of subject norms in informing consumers’ intention (Mucinhato et al., 2022; German et al., 2022), confirming the impact of subject norms on consumers’ online takeout purchase behavior. Finally, the development of network technology and the competition between network platforms are making online ordering platforms more and more personalized, entertaining, and easier for consumers to operate, reducing the cost of online ordering while increasing the residual value of consumers (Guo et al., 2021a, 2021b; Koch et al., 2022). Hence, the PBC of consumers is constantly improved, encouraging the purchase of takeout.

Our results also show that PPRP has a negative effect on consumers’ attitudes (H4), subjective norms (H5), PBC (H6), and purchase intention (H7). Among these variables, consumers’ PBC appears to be the most affected, followed by subjective norms and attitudes. This is due to the fact that the packaging pollution risk caused by buying takeout outweighs its time- and cost-saving effectiveness, thus reducing consumers’ perception of control. This conclusion supports prior observations about PBC related to package carriers (German et al., 2022) and waste sorting (Lou et al., 2022). Also, with increasing environmental protection and sustainable consumption awareness, people are compelled to reduce their purchase of takeout and use eco-friendly packaging (Koch et al., 2022). Therefore, the subjective norms surrounding online takeout purchase is weakened by PPRP. It cannot be denied that takeout meets the basic dietary needs of consumption. With a faster pace of life in the network era, takeout saves people’s time and improves their quality of life, resulting in a growing preference for takeout over cooking (Kumar et al., 2021; Kumar and Shah, 2021). However, the environmental pollution caused by takeout packaging, especially its harm to human health, contradicts the positive attitude towards takeout. Consequently, PPRP weakens consumers’ attitudes and purchase intentions towards online takeout, which is in line with prior findings, such as the association of sustainable knowledge with sustainable behavior through sustainable attitude (Walsh and Dodds, 2022).

Moreover, we found that consumers’ online takeout attitude (H8), subjective norms (H9), and PBC (H10) play a partial mediation role in the impact of PPRP on purchase intention, as the total indirect effect was found to be greater than the direct effect. Therefore, the impact of online takeout packaging pollution risk on consumers’ purchase intention acts through the mechanisms of their attitude, subjective norms, and PBC. To guide conducive environmental protection and sustainable consumption, it is necessary to reduce consumers’ purchase intention by strengthening the mediating impact of attitude, subjective norms, and PBC. The finding is aligned with prior researchers’ results that unveiled the mediating role of attitude, subject norms, and PBC in other contexts, e.g., household food-safety practices (Mucinhato et al., 2022), packaging carrier (German et al., 2022), waste sorting (Lou et al., 2022). Since attitude was found to exert the largest impact on purchase intention, more attention should be paid to shaping consumer attitudes in the process of promoting sustainable food consumption. It should be highlighted that though online takeout is fast and convenient, it comes with great potential health risks, such as microplastics (Liu et al., 2019; Prata, 2018; Ali et al., 2021), plasticizers (Freire et al., 2006; Ringbeck et al., 2022), and other additives (Segovia‐mendoza et al., 2022) in plastic packaging are harmful to the human body. In addition, the rate of recycling of plastic takeout packaging cannot keep up with the rapidly increasing rate of takeout orders (Liu et al., 2022). Substantial amounts of plastic packaging severely pollute the ecological environment and adversely impact human health (Wang and He, 2021; Xie et al., 2021). Therefore, strengthening consumers’ awareness and attitude towards takeout packaging pollution is conducive to influencing their subjective norms, such that they might influence each other to jointly reduce the purchase of takeout.

In terms of the moderating role of education levels, the impact of PBC on PI varied (H11c) across EL1 and EL3, indicating the higher education group (*β* = 0.201) is less influenced than the lower counterpart (*β* = 0.206), though the influences for both groups are not very encouraging. That is, people with higher education groups are more inclined to hold their own opinion towards their perceived behavioral control and are more objective to analyze outer conditions than the lower one. The result is in tandem with the previous studies (e.g., Ruby et al., 2019), while contradicting the result of Hwang and Choi (2019), showing the importance of contexts for the conditional effect of education level. Similarly, for the lower education group vs. medium one, the influences of PPRP on attitude (H11d) (*β* = −0.420 vs. *β* = −0.398), subjective norms (H11e) (*β* = −0.460 vs. *β* = −0.407), and PBC (H11f) (*β* = −0.487 vs. *β* = −432) are reinforced. The results confirmed again that lower-educated people are more prone to being influenced by outer circumstances (Ruby et al., 2019). Meanwhile, the results are in line with the prior research (e.g., Li et al., 2022) which highlighted consumers with higher education are more likely to motivate online food buying behavior when confronting inconvenient conditions against going outside to dine. Interestingly, concerning the association of PPRP with attitude, the higher education group (*β* = −0.456) is more impacted compared with the medium one (*β* = −0.398), which is consistent with the previous literature indicating the well-cultured consumers are inclined to more sensitive and change their actions accordingly (Oribe-Garcia et al., 2015; Han et al., 2018; Li and Wang, 2022). Taken together, the interacting influence of PPRP and education level on attitude is supposed to be considered in a more comprehensive manner rather than simply divided into lower and higher education levels. Furthermore, given the suggestion of Yagmur and van de Vijver (2012) on the association of education levels with attitudes, it is reasonable to infer that the PPRP under the circumstances of COVID-19 is a more popular and accepted perspective for higher and lower education groups than its medium counterpart.

### Theoretical implications

The development of the Internet, especially the reform of mobile networks brought by intelligent devices, is indeed a double-edged sword; it facilitates consumption, but also brings major risks. This study integrated the TPR and the TPB to design and empirically validate a model of consumers’ planned behavior toward online takeout purchases. The model depicts the trade-off between the benefits and costs of consumer decision-making under the mobile network, which has theoretical significance for the development of sustainable consumption in the post-epidemic era.

To begin with, in view of the new economic problems emerging from the COVID-19 pandemic, this study expands the connotation of risk perception (Bauer, 1960; Cox, 1967) to the consumption of online takeout, which brings convenience to life yet threatens the environment and health. The present study tested four aspects of the risk perception of takeout packaging pollution based on the literature, in-depth interviews, and questionnaire data, thereby laying a foundation for scholars to continue studying the risk of packaging pollution. Concurrently, it provides ideas for risk perception in environmental protection to be extended to other scenarios.

Furthermore, by researching the context of online takeout, we have diversified the applicability of the TPB. While the TPB has been proven in various settings (Mucinhato et al., 2022; German et al., 2022; Lou et al., 2022).), the development of technology, economy, social culture, and politics calls for further refinement of the theory’s application (Yang et al., 2022). In this regard, online takeout promotes the integration of online and offline consumption and is a vital breakthrough for studying the interaction and integration of network and physical consumption (Cheah et al., 2022). This study has taken this opportunity to illustrate the effectiveness of the TPB in takeout purchases, thus offering insights to further expand its application in online and offline integration consumption settings.

Lastly, this research deepens the relationship between the TPB and perceived risk, thereby improving our understanding of the mediation and moderation mechanisms affecting online takeout purchases. With respect to the antecedents of the TPB, many scholars have expanded the model from a positive perspective (Mucinhato et al., 2022; German et al., 2022; Lou et al., 2022). However, consumers’ decision-making is the result of the trade-off between benefits and costs, such that negative inhibitory factors must also be considered (Dominici et al., 2021; Faqih, 2022). Accordingly, this study incorporated the perceived risk of online takeout packaging as the cost factor affecting consumption and the antecedent factor of the TPB, and took into account the conditional factor of education level, which better explains its comprehensive impact on consumer behavior, improves theoretical prediction power, and provides cues for further examination of the antecedents of planned behavior.

### Practical implications

For consumers, online takeout platforms provide catering convenience. On the business side, merchants provide online catering services not only to reduce unnecessary physical store costs but also to attract more consumers through the online purchase channel (Cheah et al., 2022). However, monitoring contamination in online catering packaging is more difficult (Liu et al., 2020). The abuse of non-degradable and unhealthy packaging for online takeout entails a negative impact on the surrounding ecological environment (Schuermann and Woo, 2022). The irregular recycling and processing of online takeout packaging further cause significant pollution (Xie et al., 2021). These outcomes subsequently increase consumers’ risk perception. In particular, the hazards of takeout packaging to human health directly strengthen consumers’ risk perception, consequently reducing their purchase intentions through diminished attitudes, subjective norms, and PBC. Therefore, drastic measures should be taken to avoid this unfavorable influence mechanism.

First, from the perspective of national administration, food packaging laws and regulations need further improvement. The government should formulate supervision standards for takeout packaging at the legal and regulatory levels (Liu et al., 2020). In other words, the recycling and classification methods of packaging waste should be specified clearly to facilitate the food packaging recycling process (Govindan et al., 2022). These regulations are able to be tested in certain areas, especially in urban centers where online takeout demand is more concentrated (Wang and He, 2021), and then gradually introduced to a wider range of areas.

Second, a market adjustment mechanism needs to be built. It is urgently necessary to establish a package disposal fee system, as it is beneficial in promoting food packaging recycling efficiency. At the same time, it is imperative to reduce taxes or provide financial incentives for packaging manufacturers who use biodegradable and other environment-friendly raw materials (Stoica et al., 2020).

Third, the industry players in the online takeout supply chain should be more self-disciplined. For one, packaging manufacturers should bear in mind their social responsibility (Meena and Kumar, 2022) and strive to make breakthroughs and innovations in packaging technology to produce low-cost environmentally protective packaging, such as reusable packages (Schuermann and Woo, 2022) and degradable packages made from agriculture cellulosic waste, thereby turning trash into treasure (Ma et al., 2022). Furthermore, online takeout enterprises should give priority to degradable packaging and avoid excessive packaging. Finally, packaging recycling organizations must adopt a reasonable way, e.g., utilization of natural wastes (Hosen et al., 2022), to deal with food packaging. For instance, the best places to dispose of degradable packages are composting plants or landfills. The incineration of food packaging waste should be avoided as harmful gases are produced.

Fourth, consumers should heighten their environmental protection awareness and form scientific consumption attitudes toward takeout. The government needs to strengthen civic education to guide citizens to change their lifestyles, develop good garbage disposal habits, prioritize buying environment-friendly packaged takeout, and comply with garbage classification regulations (Govindan et al., 2022; Lou et al., 2022), especially for higher and lower educated people. This would achieve the goal of reducing the negative impacts of takeout packaging on the environment and ultimately promote sustainable consumption.

## Conclusion and future research directions

Under the pressure of the COVID-19 pandemic, retailers from various industries have had to adjust their marketing strategy to comply with emerging economic trends, which have greatly shaped consumers’ purchase patterns. Specifically, consumers now show deeper concern about their health and their consumption impacts on the environment due to the challenges faced by limited resources and unlimited commodity demands. Therefore, consumers tend to look at the pros and cons of products provided by retailers, which calls for researchers to be more aware of the factors that influence consumers’ purchase trade-off mechanisms. Employing an integrated model of perceived risk and the TPB, the current study contributes to the extant body of knowledge by illustrating how and when the perceived risk of online takeout packaging pollution affects consumer purchase intention. Our findings supply meaningful implications for future online takeout retailers. Theoretically, the mechanism underpinning online takeout purchase intention should be considered comprehensively by retailers in terms of technological strengths and environmental weaknesses. Practically, it is evident from the conclusions drawn by this study that takeout retailers in China should improve their food packaging instead of only promoting their food quality and convenience. Consumers are now more worried about the negative effects of food packaging on their health and living environment, which in turn weakens the positive impact of planned behavior on buying takeout online.

In this study, there were some limitations that still need further exploration to expand theoretical implications and applications. First, the extension of research objects is an inevitable requirement for the adaptive development of research (Ajzen, 1991). Based on the investigation of Chinese consumers, this study puts forward suggestions for the sustainable development of online takeout purchases in China, which can be used as a reference for other countries. However, given the cross-cultural differences in consumers’ perceived risk of food packaging, such as developing vs. developed countries (Tyrväinen and Karjaluoto, 2022) or collectivistic vs. individualistic cultures (Huang et al., 2022), the adaptability of the research results across cultures and countries should be addressed in future studies. Second, this study was based on the risk perception of the environmental and health hazards of online takeout, which could be extended to incorporate more detail factors and higher-order concepts to improve the model’s predictive ability. In addition to the risk factors mentioned in the study, it is worth exploring other factors (Mitchell, 1999; Molloy et al., 2022) affecting consumer behavior from a wide range of disciplines in the future. Third, although a number of extant literature works underscore the significant effect of the TPB on human behavior (Leeuw et al., 2015; Aschale et al., 2021; Bagheri et al., 2021), consumers’ attitudes, subjective norms, and PBC may be changed by long-term and external influencing perspectives, such as the risk factors that concern people in the COVID-19 pandemic situation. Thus, longitudinal factors (e.g., the data in the online takeout platform on the selecting choice of pro-environmental packaging or disposable tableware) are also worth investigating to test the actual impact on purchasing behavior rather than its intention in order to further explore the intention–behavior gap.

## Data Availability

The datasets generated during and/or analyzed during the current study are available from the corresponding author on reasonable request.
